# Inhibition of Soluble Tumor Necrosis Factor Ameliorates Synaptic Alterations and Ca^2+^ Dysregulation in Aged Rats

**DOI:** 10.1371/journal.pone.0038170

**Published:** 2012-05-29

**Authors:** Diana M. Sama, Hafiz Mohmmad Abdul, Jennifer L. Furman, Irina A. Artiushin, David E. Szymkowski, Stephen W. Scheff, Christopher M. Norris

**Affiliations:** 1 Graduate Center for Gerontology, University of Kentucky, Lexington, Kentucky, United States of America; 2 Sanders-Brown Center on Aging, University of Kentucky, Lexington, Kentucky, United States of America; 3 Molecular & Biomedical Pharmacology, University of Kentucky, Lexington, Kentucky, United States of America; 4 Xencor, Inc, Monrovia, California, United States of America; 5 Anatomy and Neurobiology, University of Kentucky, Lexington, Kentucky, United States of America; Emory University, United States of America

## Abstract

The role of tumor necrosis factor α (TNF) in neural function has been investigated extensively in several neurodegenerative conditions, but rarely in brain aging, where cognitive and physiologic changes are milder and more variable. Here, we show that protein levels for TNF receptor 1 (TNFR1) are significantly elevated in the hippocampus relative to TNF receptor 2 (TNFR2) in aged (22 months) but not young adult (6 months) Fischer 344 rats. To determine if altered TNF/TNFR1 interactions contribute to key brain aging biomarkers, aged rats received chronic (4–6 week) intracranial infusions of XPro1595: a soluble dominant negative TNF that preferentially inhibits TNFR1 signaling. Aged rats treated with XPro1595 showed improved Morris Water Maze performance, reduced microglial activation, reduced susceptibility to hippocampal long-term depression, increased protein levels for the GluR1 type glutamate receptor, and lower L-type voltage sensitive Ca^2+^ channel (VSCC) activity in hippocampal CA1 neurons. The results suggest that diverse functional changes associated with brain aging may arise, in part, from selective alterations in TNF signaling.

## Introduction

The cytokine tumor necrosis factor-alpha (TNF) plays a critical role in coordinating and maintaining immune/inflammatory responses both inside and outside the brain. TNF binds to two distinct membrane receptor subtypes, TNFR1 and TNFR2, which are, in turn, coupled to distinct intracellular signaling cascades. TNFR1 contains a classic “cytoplasmic cell death” domain and is most commonly implicated in pathological processes, while TNFR2 (which lacks the death domain) preferentially engages pathways that promote cell survival [1]. Aging and several neurodegenerative diseases are associated with elevated brain levels of TNF [2], [3], [4], [5], [6]. In animal models of disease, TNF appears to be a key contributor to chronic glial activation and impaired neuronal viability through its actions on TNFR1 [7]. However, the role of TNF and its receptor mechanisms in aging brain remain unclear.

In contrast to most disease models, aged animals exhibit neurologic changes that are generally milder and more variable in nature. These changes usually include synaptic dysfunction and Ca^2+^ dysregulation [8], [9], both of which can be precipitated in healthy young adult animals and/or in neuronal cultures in response to artificial elevations in TNF [10], [11], [12]. Perhaps most relevant to the aging neurologic phenotype is evidence from neuronal culture studies showing that TNF potentiates the activity of L-type voltage sensitive Ca^2+^ channels (L-VSCCs) [11]. A similar potentiation of L-VSCC activity has been well-characterized in hippocampal neurons of memory impaired aged rats [13] and is a primary mechanism for alterations in short and long-lasting forms of synaptic plasticity [9].

While a recent behavioral study in aged rats showed that TNF blockade in the cerebellum accelerated learning in a delayed eyeblink task [14], no studies that we know of have directly investigated the impact of TNF on synaptic and Ca^2+^ signaling mechanisms during aging. Here, we report that protein levels for the major TNFRs are altered in the hippocampus of aged Fischer 344 rats, in favor of TNFR1 signaling. Furthermore, selective blockade of TNF/TNFR1 interactions in aged rats over a four-to-six week period using a novel anti-TNF biologic (XPro1595) improved behavioral performance on a Morris swim task, reduced microglial activation, prevented the induction of hippocampal long-term depression (LTD), and reduced the activity of L-VSCCs in CA1 neurons. Together, the results suggest that TNF/TNFR1 interactions play an important role in shaping the neurologic phenotype of aged animals and could set the stage for further pathological changes associated with neurologic disease.

## Methods

### Ethics Statement

All animal procedures were compliant with the guidelines of the University of Kentucky institutional Animal Use Committee and the American Association for Accreditation of Laboratory Animal Care.

### Animals

Studies used six- or twenty-two-month-old Fischer 344 rats (National Institute on Aging) which were housed separately, provided *ad libitum* food and water, and maintained on a 12 h∶12 h light∶dark cycle.

### Surgeries

Rats were anaesthetized with isoflurane (2.5%) and immobilized in a stereotaxic frame. Depending on the study, cannulae were inserted unilaterally into the right lateral ventricle (from Bregma −1, +1.4 mm lateral), or bilaterally into the hippocampus (from Bregma, −3.8, ±2 mm lateral). Cannulae were connected to osmotic pumps (Alzet, Model 2004 or Model 2006 for four and six week delivery periods, respectively) inserted subcutaneously behind the shoulders. Rats that showed weight loss of more than 10 grams after surgery were provided a high-calorie food supplement until their weight stabilized. If other problems were discovered, which were rare and usually not related to surgery, treatment recommendations from the veterinarian staff were followed. Osmotic pumps were charged with either vehicle or XPro1595 (0.08 mg/kg/day), a novel dominant-negative TNF (formerly XENP1595 [15]) manufactured by Xencor. XPro1595 selectively inhibits soluble TNF (solTNF) signaling by exchanging subunits with solTNF trimers for subunits with disrupted receptor binding surfaces, therefore preventing interaction with TNF receptors [16], but primarily inhibiting TNFR1 which has much greater affinity for solTNF [17]. Transmembrane TNF (tmTNF) function is spared by XPro1595 therapy, maintaining the protective roles of TNF which occur primarily through TNFR2 signaling [15]. Though initially developed for targeting TNF signaling in peripheral tissues, Xpro1595, or similar compounds from Xencor, have been shown to inhibit TNF-mediated activation of NFκB in a neural cell line [18] and prevent microglial activation in primary murine cell cultures [18] and in intact animal models of Parkinson's disease [19], suggesting that XPro1595 should effectively inhibit TNF signaling in the hippocampus of aged rats.

### Morris Water Maze

The water maze was a black circular pool (183 cm in diameter) filled with water heated to 27°C. Each subject's path in the pool was recorded with Videomex Water Maze Monitoring Software (Columbus Instruments, Columbus, OH) and analyzed off-line. All animals were first screened for visual and/or motor impairments using a visual cue version of the water maze [20]. Two days after the cue task, rats received training on a spatial version of the Morris Water Maze according to methods previously described by our group [20], [21]. The all-black escape platform, located about 1.5 cm below the water's surface, remained in the same quadrant across trials for each group of animals, but varied from group to group. The curtains surrounding the pool were removed, revealing extra-maze cues within the room, including high-contrast, black and white poster boards. Starting locations were randomized across trials. Training consisted of six blocks of three trials (60 s/trial) with an inter-trial interval of 60 s and an inter-block interval of 20–30 min. Latency and path length traveled to escape were used to measure performance on each trial.

### Synaptic strength and plasticity measures in intact hippocampal slices

Methods for preparation of hippocampal slices are similar to that described previously by our group [22]. Four to fourteen days after behavioral assessment, rats were anesthetized deeply with CO_2_ and then decapitated. Brains were removed rapidly and stored briefly in Ca^2+^-free chilled, oxygenated (95% O_2_/5% CO_2_) artificial cerebrospinal fluid (aCSF) before hippocampi were dissected. The composition of aCSF was (in mM): 124 NaCl, 2 KCl, 1.25 K_2_PO_4_, 2 MgSO_4_, 2 CaCl_2_, 26 NaHCO_3_, 10 dextrose, pH = 7.4. Hippocampal slices (450 µm), cut parallel to the alvear fibers, were prepared with a McIlwain tissue chopper and transferred to a four-well humidified interface holding chamber containing heated (30–32°C) and oxygenated aCSF with 2 mM CaCl.

After 1–1.5 hours, slices were transferred to a modified RC-22 recording chamber (Warner Instruments) and perfused continuously (1–1.5 ml/min) with oxygenated aCSF heated to 32°C. A bipolar platinum-iridium stimulating electrode, positioned in *stratum radiatum* near the CA3 border, was used to deliver 100 µsec diphasic, constant-current pulses to CA3 Schaffer collaterals. Stimulus intensity was controlled by a constant current stimulus isolation unit (World Precision Instruments). Stimulus timing was controlled by a Multiclamp 700B amplifier and pClamp software (Molecular Devices). A glass micropipette (1–6 MΩ), pulled from thin-wall tubing (Drummond Scientific Company, 100 µl calibrated pipets), filled with aCSF and containing a Ag/AgCl wire, was positioned about 1 mm from the stimulating electrode in *stratum radiatum* of CA1. Field excitatory postsynaptic potentials (EPSPs) were amplified 100X, Bessel-filtered at 1 kHz, and digitized at 10 kHz.

For each slice, stimulus pulses were delivered at nine different intensity levels (range 30–500 μA) at a rate of 0.1 Hz to establish a synaptic strength curve. Five field potentials at each stimulus level were averaged, and measurements of fiber volley (FV) amplitude (in mV) and EPSP slope (in mV/ms) were performed offline using pClamp software (Molecular Devices). The relative number of activated CA3 afferents in each slice was assessed by plotting average FV amplitudes against stimulation intensity. Averaged EPSP slope measures were then plotted against their corresponding FV amplitudes to estimate the strength of available CA3-CA1 synaptic contacts. Fiber excitability and synaptic strength curves for each hemisphere were fit with a sigmoidal equation of the form: a/(1+exp(−(x−x0)/b)), where *a* equals the maximal amplitude of the distribution, *b* equals the distribution slope, and x0 equals the stimulation intensity (or FV amplitude) required for half-maximal response amplitude. Fitted parameters were then compared across treatment groups using Z tests. Z values greater than |2| were considered significant. After the synaptic strength curve, stimulation intensity for each slice was readjusted to elicit an EPSP of ∼1 mV. Stimulus pulses were then delivered at 0.033 Hz until a stable 20 min baseline was established. Low-frequency stimulation (LFS; 900 pulses at 1 Hz) was delivered to induce long-term depression (LTD), followed by an additional 60 min baseline.

For each animal, electrophysiological parameters were averaged across all slices (usually one to three slices) and the *n* used for all statistical comparisons reflects the number of animals per drug treatment group. Within each treatment group, EPSP slope measurements from the last 10 min of the post-LTD baseline were averaged across slices and compared to the pre-LTD baseline slope average. Differences across groups were determined by repeated measures ANOVA, with significance at *p*<0.05.

### L-VSCC activity measurements in hippocampal zipper slices

Acute hippocampal slices were prepared from rats as described above and then partially dissociated along the CA1 cell body layer to create “zipper” slices as described previously [13], [23], [24], [25], [26]. Immediately before electrophysiologic recordings, each slice was nicked with a scalpel blade at the CA1 cell body layer and transferred to a small microcentrifuge tube containing approximately 1 ml of Ca^2+^-free aCSF with 10 mM EGTA. Slices were then shaken gently by hand and monitored periodically for dissociation along CA1 *stratum pyramidale* before transfer to a perfusion-style recording chamber (Warner Instruments) containing recording medium. A 40x objective was used to identify cleanly exposed CA1 pyramidal neurons, suitable for recording. On-cell patch extracellular recording solution contained (in mM): 140 K gluconate, 3 MgCl_2_, 10 glucose, 10 EGTA, and 10 HEPES, pH 7.35, osmolarity  = 300 mOsm. On-cell patch pipette solution consisted of (in mM): 20 BaCl_2_, 90 choline Cl, 10 TEA Cl, and 10 HEPES, pH 7.35, osmolarity  = 290 mOsm.

On-cell recording patch pipettes consisted of fire-polished, Sylgard-coated, glass capillary tubes with tip resistances averaging 5.89±0.07 MΩ across groups. All recordings were obtained at room temperature using an Axopatch 200A patch-clamp amplifier and pClamp software (Molecular Devices). Seal quality was determined using the seal test feature of pClamp. Patches that sealed slowly (>30 sec) or showed a seal resistance <10 GΩ were excluded from statistical analysis. A total of 25 patches for vehicle-treated rats and 28 patches for XPro1595-treated rats were used for statistical analysis. Maximal current in each patch was achieved by stepping the patch membrane from its holding potential of −70 to +10 mV. A series of 45−50 steps to +10 mV (15 s inter-step interval) was used to generate an average ensemble current for each patch. Currents were leak-subtracted off-line using hyperpolarizing pulses, identical in duration and opposite in polarity. Current density (pA/μm^2^) for each patch was derived by dividing the average ensemble current by the patch area. Patch area, which is inversely proportional to the pipette resistance, was estimated for each recording using the equation *a* = 12.6(1/*R* + 0.018), where ‘*a*’ is the patch area and ‘*R*’ is the pipette resistance [27]. Data were filtered at 2 kHz and digitized at 5 kHz.

**Figure 1:**
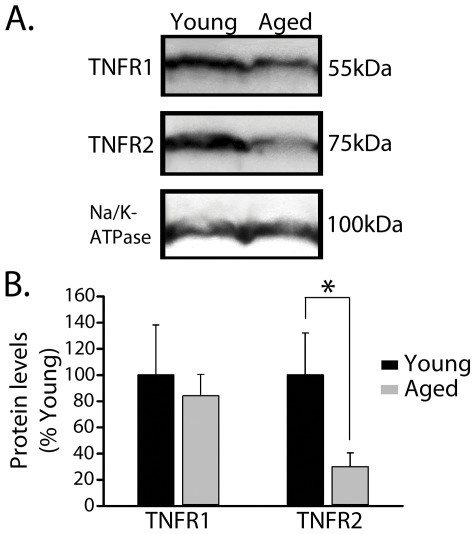
Increased TNFR1/TNFR2 ratio during aging. ***A,*** Representative Western blots for TNFR1 and TNFR2 in hippocampal membrane fractions from young (6 mos; *n* = 6) and aged (22 mos; *n = *10) male Fischer rats, with the Na/K-ATPase loading control shown below. ***B,*** There was no significant change in TNFR1 protein levels with age (*p* = 0.67); however, there was a significant decrease in TNFR2 with age (* *p*<0.05).

### Measurement of hippocampal proteins

For each rat, one hippocampus was flash-frozen in liquid nitrogen and stored at −80°C for later use. Cytosolic and nuclear fractions from hippocampal samples were prepared with modifications of the protocol used by Ohlsson and Edlund [28]. Briefly, the thawed hippocampus was homogenized in buffer A [25 mM HEPES, pH 7.0, 25 mM KCl, 5 mM MgCl_2_, 0.05 mM EDTA, 10% glycerol, 0.1% NP-40, and 1 mM dithiothreitol (DTT), phosphatase inhibitor, protease inhibitor, calpain inhibitors] and centrifuged for 15 min at 3000 rpm (4°C). The resulting pellet was resuspended in Buffer C [50 mM HEPES, pH 7.6, 50 mM KCl, 0.1 mM EDTA, 10% glycerol, 1 mM dithiothreitol, phosphatase inhibitor, protease inhibitor, calpain inhibitors, and 0.3 M ammonium sulfate], incubated on a rocking platform for 30 min (4°C) and then ultracentrifuged at 37,000 rpm for 15 min at 4°C to remove nuclear debris. Proteins in the supernatant were precipitated by adding an equal volume of 3 M ammonium sulfate, incubated on a rocking platform for 15 min at 4°C, and ultracentrifuged at 37,000 rpm for 15 min. The pellet obtained, which contained the nuclear fraction, was resuspended in 150 µl of buffer C and stored at –80°C. The supernatant collected from the initial 3000 rpm centrifugation step described above was processed through a series of three 15 min ultracentrifugation (37,000 rpm) steps. After the first spin, the pellet obtained contained the membrane fraction and was resuspended in 200 µl of 0.3 M sucrose buffer and stored at –80°C until use. Then, buffer B [0.3 M HEPES, pH 7.6, 50 mM KCl, 0.1 mM EDTA, 1 mM DTT, phosphatase inhibitor, protease inhibitor, calpain inhibitors] was added to the supernatant at 1/10^th^ the volume and spun a second time. The resulting supernatant was combined with 0.3 g/ml ammonium sulfate, rotated for 30 min at 4°C for protein precipitation, and then spun a third time. The resulting pellet, which contained the cytosolic fraction, was resuspended in 200 µl of buffer C and stored at –80°C until use.

Protein levels of the membrane and cytosolic fractions were estimated using the Lowry method. Samples were loaded into individual lanes of Bio-Rad pre-cast gels (7.5% or 4–20% gradient), or into freshly cast 10% SDS-PAGE gels with protein concentrations held constant across lanes. Proteins were resolved by electrophoresis and transferred to polyvinylidene difluoride membranes for semi-quantitative Western blot. Membranes were incubated at 4°C overnight in 5% non-fat milk along with primary antibodies, which included mouse anti-NR1 1∶2000 (Millipore), rabbit anti-NR2A 1∶3000 (Millipore), rabbit anti-NR2B 1∶2000 (Millipore), rabbit anti-GluR1 1∶2000 (Millipore), anti-GluR2 1∶1000 (Santa Cruz Biotechnology), rabbit anti-Ca_V_1.2 1∶2000 (Alamone Labs), rabbit anti-Ca_V_1.3 1∶1000 (Alamone Labs), mouse anti-GFAP 1∶10,000 (Cell Signaling Technology), goat anti-TNFR1 1∶250 (Santa Cruz Biotechnology), goat anti-TNFR2 1∶250 (Santa Cruz Biotechnology), mouse anti-NaKATPase 1∶10,000 (Abcam), and mouse anti-GAPDH 1∶10,000 (Abcam). Primary antibodies were tagged with an appropriate horseradish peroxidase-conjugated secondary antibody, diluted in 5% non-fat milk at 1∶10,000, and detected using the ECL-plus Western kit (GE Healthcare). Protein levels were quantified using a Storm 860 molecular imager.

**Figure 2:**
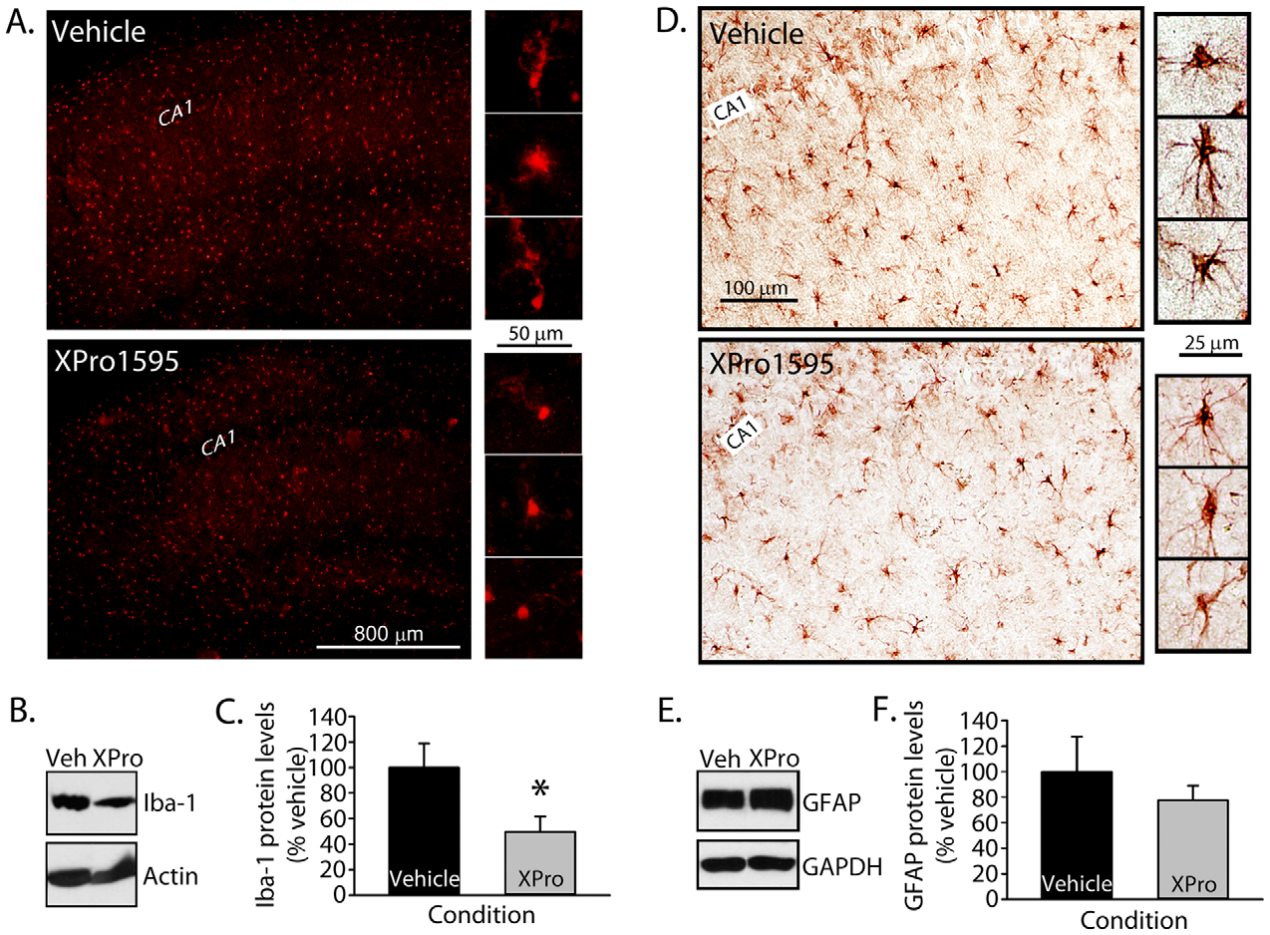
XPro1595 alters Iba-1 levels. XPro1595 (0.08 mg/kg/day) was administered to the hippocampus over a six week period using Alzet osmotic pumps. Immunolabeling revealed a reduction in inflammatory phenotype in microglia (***A*** **, Iba-1**) and, to a lesser extent, in astrocytes (***D*** **, GFAP**) of XPro1595 treated aged rats. CA1 = CA1 *stratum pyramidale*. Representative Western blots and mean ± SEM protein levels are for Iba-1 (***B*** and ***C***) and GFAP (***E*** and ***F***) in hippocampal fractions of vehicle and XPro1595-treated rats. β-actin and GAPDH served as loading controls for Iba-1 and GFAP, respectively. Results revealed a marked, significant reduction in Iba-1 protein levels (* *p*<0.05) and a smaller, nonsignificant reduction in GFAP levels in the XPro1595 group (*p* = 0.45).

### Immunohistochemistry

Some hippocampal slices processed for electrophysiologic recordings were subsequently used for immunohistochemical labeling of microglia (with Iba-1) and astrocytes (with GFAP). Slices were fixed in 4% paraformaldehyde overnight, preserved in sucrose buffer, and further cut to 50 µM thickness using a freezing microtome. Sections were mounted on gelatin-coated slides and baked in an oven at 37°C for one week. Slices were rehydrated through a series of ethanol washes to water before heat-induced antigen retrieval (Diva Decloaking solution from Biocare Medical) was performed.

For Iba-1 labeling, slide-mounted sections were first blocked for 1 hr at room temperature with phosphate buffer saline (PBS) containing 10% donkey serum and then incubated overnight at 4°C with primary antibody, 1∶500 rabbit anti-Iba-1 (Wako) in PBS. Slides were washed three times with PBS and incubated for 2 h at room temperature with the secondary antibody, Alexa Fluor 594 1∶500 donkey anti-rabbit (Jackson Immunoresearch Laboratories, Inc) diluted in blocking buffer. After rinsing three times with PBS, sections were dehydrated, via a series of ethanol washes, rinsed in 100% xylene and coverslipped with Prolong gold anti-fading mounting reagent (Invitrogen). Cell fluorescence was imaged with an inverted epifluorescence confocal microscope (DMIRE-2, Leica).

For GFAP labeling, slide-mounted sections were first blocked for 1 h at room temperature with Tris buffered saline (TBS) containing 15% horse serum and then incubated overnight at 4°C with primary antibody, 1∶50 mouse monoclonal anti-GFAP (Cell Signaling Technology Inc) in TBS. Slides were washed three times with TBS and incubated for 2 h at room temperature with the secondary antibody, 1∶200 biotinylated horse anti-mouse (Vector Laboratories, Inc) diluted in TBS. Sections were rinsed three times with TBS and then incubated in ABC reagent (Vectastain ABC Standard Kit, Vector Laboratories, Inc) for 1 h before being developed with Nova Red, and then finally dehydrated, washed in 100% xylene, and coverslipped with mounting medium (Richard-Allen Scientific). Sections were imaged with a Nikon Eclipse E800.

### Statistics

Student's t-test, with significance set at *p*<0.05, was used to analyze Western blot protein levels, and cell-attached patch “zipper” L-VSCC current density. Repeated-measures analysis of variance (rm-ANOVA), with significance set at *p*<0.05, was used to analyze spatial learning in the Morris Water Maze, and the extent of synaptic depression following the delivery of 1 Hz stimulation. Z-test, with significance set at *p*<0.05, was used to analyze the 3-parameter sigmoidal functions that were fit to the fiber excitability and synaptic strength curves.

## Results

### Increased TNFR1/TNFR2 ratio during aging

TNF levels increase in the hippocampus during aging [2], [3], [4], but little is known about the relative expression levels of TNFRs, which play fundamental roles in determining the physiologic outcome of elevated TNF [29]. To investigate TNFR levels, hippocampal membrane fractions were prepared from six young (6 month) and ten aged (22 month) male rats for Western blot analysis of TNFR1 and TNFR2 (Figure 1A and 1B). The results revealed a dramatic and significant drop (∼70%) in membrane TNFR2 levels in aged rats (*p*<0.05), while TNFR1 levels were relatively unaffected by age (*p* = 0.67). Accordingly, the ratio of TNFR1 to total TNFR exhibited a significant increase in the aged group suggesting that overall TNFR availability in aged rat hippocampus favors TNFR1 signaling.

### XPro1595 reduces signs of microglial activation in aged rats

To determine the extent to which TNF/TNFR1 signaling contributes to key brain aging biomarkers, we next treated aged rats with the novel biologic XPro1595, manufactured by Xencor. XPro1595 specifically targets and inactivates soluble TNF (solTNF) trimers, but does not disrupt the function of transmembrane TNF (tmTNF) [15], [16]. As TNFR1 exhibits high affinity to solTNF, whereas TNFR2 shows greater affinity for tmTNF [17], [30], XPro1595 essentially acts as a selective inhibitor of TNFR1 signaling [1], [15], [16]. XPro1595 was infused bilaterally into the hippocampus of aged rats for six weeks using Alzet osmotic pumps. Drug concentration and delivery rate (0.08 mg/kg/day) was based on published work showing efficacy of a similar Xencor construct in an intact rat model of Parkinson's disease [19] and our pilot studies.

In models of Parkinson's disease, XPro1595 (or similar constructs) strongly suppressed signs of neuroinflammation, including the activation of microglia [19]. To determine the extent to which TNF/TNFR1 signaling drives neuroinflammation during normal aging, we labeled hippocampal sections prepared from vehicle- and XPro1595-treated (*n = *10, *n* = 10, respectively) rats for the presence of Iba-1 (marker for microglial activation) or GFAP (marker for astrocyte activation). Western blots were also used to quantitatively assess levels of these proteins in hippocampal homogenates. As shown in Figure 2A, Iba-1-labeled microglia appeared less ramified in the hippocampus of XPro1595-treated rats. Consistent with this observation, XPro1595 treatment was associated with a significant reduction (*p*<0.05) in hippocampal Iba1-protein levels as determined by Western Blot (Figure 2B, 2C). A similar trend was observed for GFAP (Figure 2D–2F), though differences between vehicle- and XPro1595-treated rats in Western analyses did not reach statistical significance (*p* = 0.45). Thus, similar to that shown in Parkinson's disease models, XPro1595 also appears to exert anti-neuroinflammatory actions during aging.

### XPro1595 accelerates behavioral acquisition, reduces age-related susceptibility to long-term depression, and increases expression of GluR1 protein

**Figure 3:**
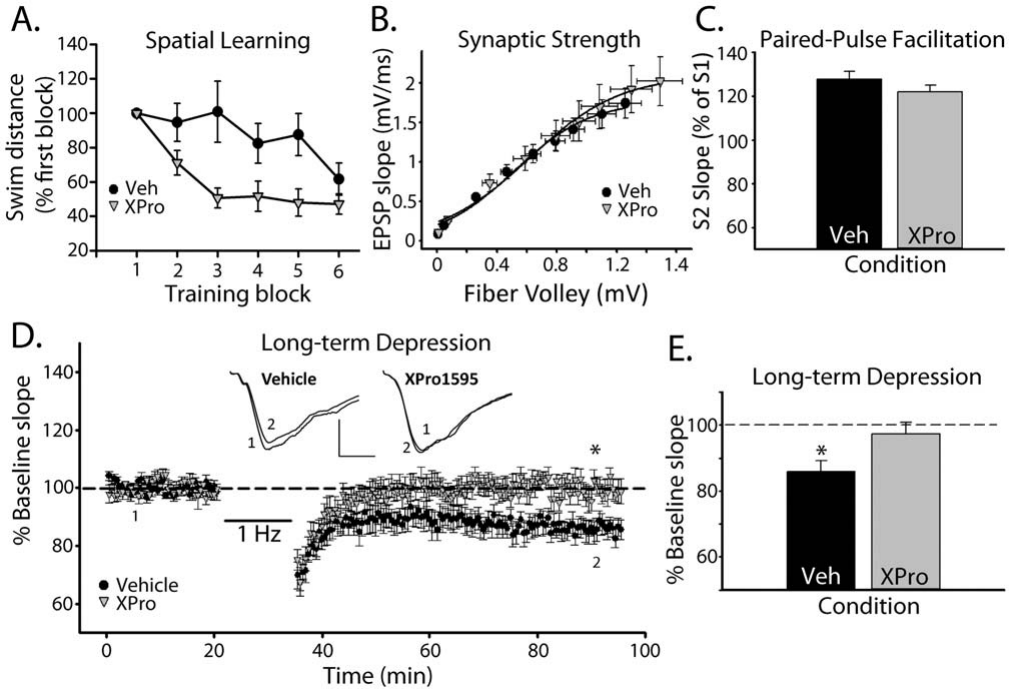
XPro1595 improves learning and synaptic measures. *A,* For behavioral assays, XPro1595 (0.08 mg/kg/day) was administered to the hippocampus over a six week period. Performance (mean ± SEM) in the spatial version of the Morris Water Maze was measured by path length (cm), and normalized (%) to block one performance levels for each rat. Each group showed significant improvement by block 6 (*p*<0.01). However, XPro1595-, but not vehicle-treated, rats showed significant improvement as early as block 2 and 3, suggesting that TNF-blockade accelerates learning rates during aging. ***B,*** Mean EPSP slope amplitudes (vertical SEM bars) were plotted against mean FV amplitudes (horizontal SEM bars) to generate CA3-CA1 synaptic strength curves. Each average curve was fit with sigmoidal equations and compared across treatment groups using Z tests. Aged rats that received intraventricular infusions of XPro1595 over a four week period (0.08 mg/kg/day) exhibited a slight, but significant increase in maximal EPSP amplitude, relative to the vehicle group (*z* = 2.17). Other curve parameters (*i.e.* slope and half-maximal FV) were not affected by XPro1595. ***C,*** In a twin pulse paradigm (50 ms interpulse interval), the EPSP slope corresponding to pulse 2 (S2) was expressed as a percentage (mean ± SEM) of the pulse 1 (S1) EPSP slope to obtain measures of paired-pulse facilitation (PPF). No treatment effect was observed for PPF (*p* = 0.27). ***D,*** Time plot showing normalized mean ± SEM EPSP slope amplitudes collected before (1) and after (2) the delivery of prolonged 1 Hz stimulation (bar, 900 consecutive pulses). The inset shows representative CA1 EPSP waveforms averaged in the pre- (1) and post-1 Hz (2) periods for vehicle and XPro1595 groups. Scale bars indicate 0.5 mV vertical by 2 ms horizontal. ***E,*** Bar graph shows the amount of LTD, expressed as a percentage of the pre-1 Hz baseline (mean ± SEM) in each treatment condition. The results revealed significant LTD in vehicle, but not in XPro1595-treated rats (* *p*<0.05, repeated measures ANOVA).

Elevated levels of pro-inflammatory cytokines, including TNF, have been suggested to negatively impact cognition and synaptic plasticity in aging and age-related neurodegenerative disease [6], [10], [31], [32], [33]. To investigate the extent to which TNF blockade via hippocampal XPro1595 infusion (0.08 mg/kg/day) influences cognitive status during aging, drug- and vehicle-treated (*n* = 11, *n = *13, respectively) rats were trained on the spatial version of the Morris Water Maze. As shown in Figure 3A, both groups of animals learned the task by block six (*p<*0.01). However, rats treated for six weeks with XPro1595 exhibited significantly faster acquisition rates (*p*<0.05) than the vehicle treated group, suggesting that blockade of solTNF signaling improves learning during aging.

Behavioral deficits in aged rats are associated with changes in synaptic strength [34] and susceptibility to induction of long-term depression (LTD) [35]. To determine if TNF/TNFR1 signaling modulates synaptic strength parameters and LTD, XPro1595 (*n* = 10) or vehicle (*n* = 8) was delivered intraventricularly to aged rats continuously (0.08 mg/kg/day) across a four week period using micro-osmotic pumps. Field potentials in CA1 *stratum radiatum* of CA1 were recorded in response to electrical stimulation of CA3 Schaffer collaterals as described previously [36], [37]. For each slice, an input/output curve was constructed from nine stimulus intensity levels. Fiber excitability (*i.e.* FV amplitude vs. stimulus intensity, not shown) and synaptic strength (*i.e.* EPSP slope amplitude vs FV amplitude, Figure 3B) data were fitted with a three-parameter sigmoidal function and compared across groups as described [38]. Results indicated no difference in slope or half-maximal amplitude in the synaptic strength curve, but XPro1595-treated rats did show a slight increase in the maximal EPSP amplitude (Figure 3B; *z* = 2.17), in the absence of a significant effect on FV amplitude (not shown). In addition to synaptic strength, measures of paired pulse-facilitation (PPF) were also relatively insensitive to XPro1595 (Figure 3C), suggesting that TNF signaling does not appreciably alter excitation-neurotransmitter release coupling in aged rats.

LTD was induced in CA1 using low frequency stimulation (1 Hz, 900 pulses) and data were analyzed using two-way repeated measures ANOVA (Figure 3D and 3E). EPSP slope magnitudes measured 60 min after 1 Hz trains were significantly reduced relative to baseline in the vehicle treated aged group (*p*<0.05), similar to previous findings [35], [36], [39], [40]. Alternatively, slices from XPro1595 treated animals showed nearly no depression (about 3%) 60 min after 1 Hz stimulation, suggesting that TNF/TNFR1 signaling is permissive for LTD induction during aging.

**Figure 4:**
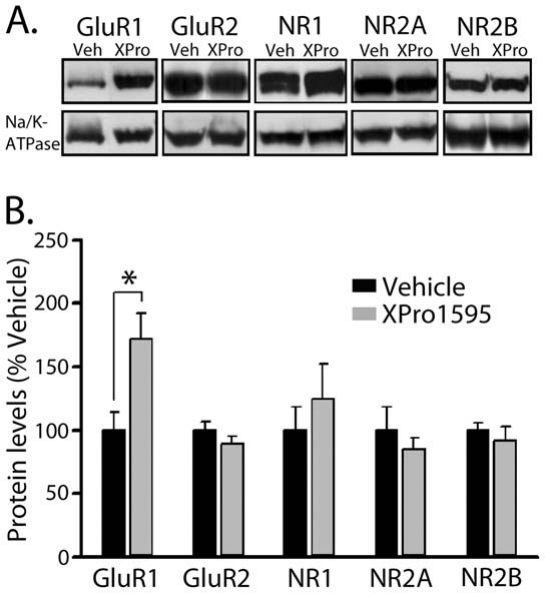
Effects of XPro1595 on glutamate receptor protein levels. *A,* Representative Western blots are shown for AMPA receptor (GluR1 and GluR2) and NMDA receptor subtypes (NR1, NR2A, and NR2B) in hippocampal membrane fractions from aged (22 month) rats treated for four weeks (intraventricular delivery) with vehicle or XPro1595 (0.08 mg/kg/day). The Na/K-ATPase loading control is shown below. ***B,*** XPro1595 treatment resulted in a selective increase in GluR1 levels (* *p*<0.05).

In addition to functional synaptic measures, we also used Western blot to assess glutamate receptor protein levels in membrane fractions from vehicle and XPro1595-treated rats. Protein markers included the AMPA receptor subunits GluR1 and GluR2, and the NMDA receptor subunits NR1, NR2A, and NR2B (Figure 4A). Nearly all of the glutamate receptor subunits investigated were insensitive to blockade of TNF/TNFR1 signaling (Figure 4A and 4B). The exception was GluR1, which showed strikingly higher membrane levels in XPro1595-treated aged rats (∼71% increase, *p*<0.05).

### XPro1595 reduces L-VSCC activity in aged rats

**Figure 5:**
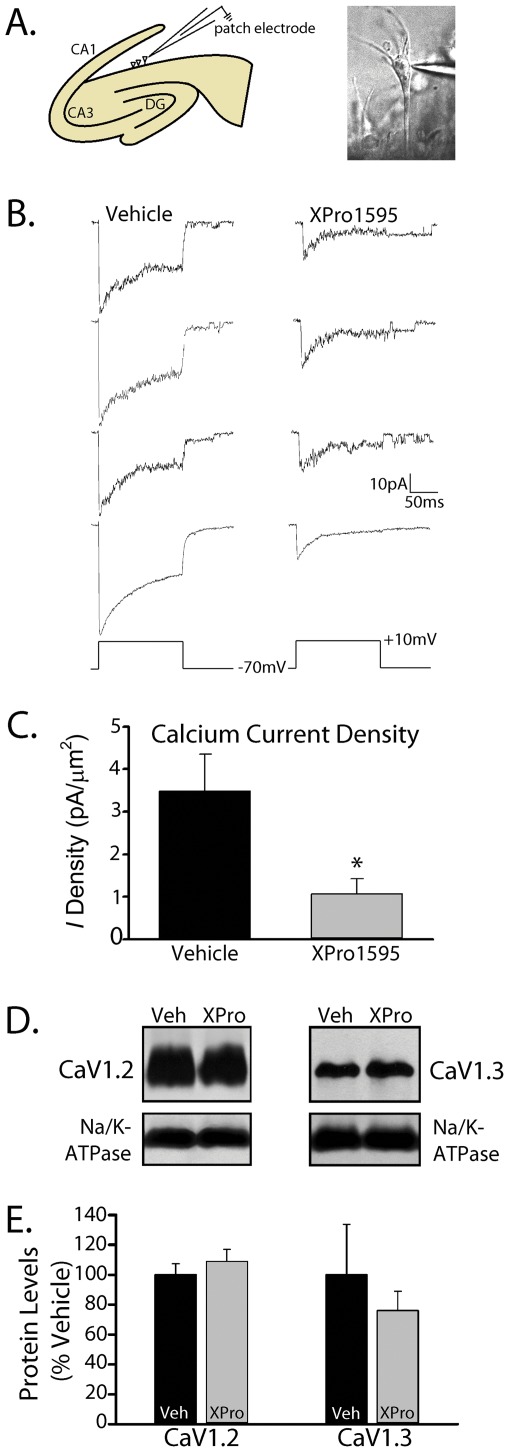
XPro1595 reduces L-VSCC activity in CA1 neurons of aged rats. *A,* * Left panel*, Cartoon illustration of a partially dissociated hippocampal slice in which CA1 is “unzipped” along *stratum pyramidale* to expose CA1 neurons for patch clamp recording. *Right panel*, Photomicrograph showing a glass micropipette tip patched onto a CA1 pyramidal neuron in an “unzipped” slice. ***B,*** Three representative L-VSCC current traces and the average ensemble current (bottom trace) in cell attached patches from aged rats treated for six weeks (intrahippocampal delivery) with vehicle or XPro1595 (0.08 mg/kg/day). Traces were taken from 45–50 consecutive step depolarizations (150 ms duration) from −70 to +10 mV. ***C,*** Mean ± SEM current (*I*) densities (pA/μm^2^) for each treatment group are shown. XPro1595 significantly reduced VSCC *I* density nearly three-fold (* *p*<0.05). ***D–E,*** Representative Western blots (***D***) and mean+SEM protein levels (***E***) for the major pore-forming L-VSCC subunits (Ca_V_1.2 and Ca_V_1.3) from hippocampal membrane fractions. The Na/K-ATPase served as loading control. XPro1595 did not significantly alter either Ca_V_1.2 (*p* = 0.17) or Ca_V_1.3 (*p* = 0.63) levels.

Age-related alterations in short and long-lasting forms of synaptic plasticity appear to be strongly linked to the activity of L-VSCCs [37], [41], which is increased in CA1 pyramidal neurons during aging [13], [25], [42], [43]. We previously reported that blockade of L-VSCCs prevents the induction of LTD in aged animals [37], much like what is seen with TNF blockade (Figure 3D). Interestingly, TNF has been shown to potentiate L-VSCC activity in neuronal cultures [11], suggesting that blockade of TNF/TNFR1 signaling may reduce L-VSCC activity in aged rats. To test this possibility, an additional cohort of aged rats received intrahippocampal perfusions of vehicle (*n* = 12) or XPro1595 (0.08 mg/kg/day; *n* = 14) across six weeks, followed by extraction of hippocampi for preparation of “zipper slices” as described [13], [25]. Cleanly exposed CA1 neurons (Figure 5A) were sealed with a glass micropipette containing recording solution and the potent L-VSCC agonist Bay-K 8644 to maximize the probability of L-VSCC openings. L-VSCC activity was recorded in cell-attached patch mode from a total of 53 cells (25 vehicle, 28 XPro1595). Maximal L-VSCC activity was elicited by step depolarizations from −70 to +10 mV, and averaged across 45–50 sweeps. Maximal peak L-VSCC current densities (pA/μm^2^ of membrane) in two or more patches from the same rat were averaged to produce a single data point for each rat. Average L-VSCC current (*I*) density (pA/μM^2^) was then compared across groups using an unpaired t-test. As shown in Figure 5C, XPro1595-treated aged rats exhibited a 3-fold decrease in cell-attached L-VSCC current density (*p*<0.05), suggesting that TNF/TNFR1 signaling potentiates, or is permissive for L-VSCC activity in aged rats.

Previous work suggested that the age-related increase in L-VSCC activity is attributable to an increase in the expression of L-VSCC Ca_V_1 pore forming subunits [44], [45], [46], particularly the Ca_V_1.3 subunit. To determine if XPro1595 effects on L-VSCC activity are associated with the reduced expression of Ca_V_1 subunits, we measured relative hippocampal membrane protein levels of Ca_V_1.2 and Ca_V_1.3 in vehicle and XPro1595-treated aged rats using Western blot. As shown in Figures 5D and 5E, neither Ca_V_1 subunit was significantly affected by TNF blockade, suggesting that the observed differences in L-VSCC activity shown in Figures 5B and 5C are likely the result of functional alterations, rather than changes in total channel protein levels. However, these data cannot rule out the possibility that the cell membrane surface expression of Ca_V_1 subunits was significantly affected by XPro1595.

## Discussion

TNF is among the key cytokines involved in initiating and maintaining neuroinflammation. Other neurologic processes, including synaptic function and plasticity, also appear to be modulated by TNF. Though elevated in the brain during non-pathologic aging, the present study is among the few to investigate the specific effects of TNF in intact aged animals. TNF primarily acts on two distinct receptor subtypes, TNFR1 and TNFR2, which are linked to common and divergent signaling pathways [1]. TNFR1 contains a classic cytoplasmic cell death domain (CDD) and is coupled to caspases involved in apoptosis, whereas TNFR2 lacks the CDD and activates PI3-kinase signaling cascades associated with cell survival. The expressional balance of TNFR subtypes appears to be critical to the function of multiple organ systems, including brain. For instance, alterations in the balance of TNFR expression, in favor of TNFR1, are associated with higher levels of excitotoxic damage in primary neurons [29].

Here, we showed for the first time that the TNFR1:TNFR2 ratio is increased nearly three-fold in the hippocampus of aged, relative to young adult rats (Figure 1), suggesting that TNF increasingly acts through TNFR1-coupled pathways during aging. We next used the novel anti-TNF biologic, XPro1595, to essentially isolate the contribution of TNF/TNFR1 interactions to neurologic function in an intact aged rats. XPro1595 is far more selective for TNF/TNFR1 signaling because it only disrupts the biological activity of soluble TNF trimers [16], which, in turn, show far greater affinity to TNFR1, relative to TNFR2 [17]. Similar to previous results obtained in neurodegenerative disease models [19], chronic intracranial infusion with XPro1595 strongly suppressed microglial activation in aged rats (see Figure 2A), confirming the anti-inflammatory actions of this drug. As discussed further below, XPro1595 also had a positive impact on multiple other functional outcome measures in aged rats. The unique pharmacologic mechanism of XPro1595 suggests that age-related neurologic dysfunction is strongly related to alterations in TNF/TNR1 signaling. Nonetheless, based on our data alone, we cannot entirely rule out the possibility that other potentially detrimental roles involving TNF/TNFR2 signaling contribute to age-related neurologic changes, since this pathway was not specifically modulated in this study.

### Effects of XPro1595 on synaptic function and plasticity in aged rats

Memory deficits in aged mammals are widely believed to result from alterations in the strength and/or plasticity of hippocampal synapses [47], [48], [49]. Though specific observations have varied across labs, attributable, perhaps, to different methodologies and experimental models [22], [50], [51], our group and others have shown that susceptibility to LTD is increased in area CA1 during aging [36], [39], [52], [53], [54]. In Fischer 344 rats, the extent of LTD in CA1 is inversely correlated with performance on the Morris swim task [35]. Moreover, several manipulations that disrupt LTD induction, including blockade of NMDARs and L-VSCCs [37], [55], [56], activation of estrogen receptors [39], [54], [57] and inhibition of protein phosphatase signaling pathways [58] have also been shown to improve cognitive function in aged rodents [20], [44], [57], [59], [60].

The present study suggests that TNF/TNFR1 signaling may also contribute to cognitive decline during aging through its effects on LTD mechanisms. Similar to a recent study on delayed eyeblink conditioning [14], chronic blockade of TNF signaling in aged rats using XPro1595 accelerated acquisition of a Morris swim task. Conversely, LTD, which was robust in CA1 of vehicle-treated aged rats, was nearly absent in the XPro1595 group. Aged rats treated with XPro1595 also exhibited a small, albeit significant, increase in the maximal evoked EPSP amplitude, suggesting that TNF/TNFR1 interactions may also have subtle effects on basal synaptic transmission parameters. Changes in synaptic function in XPro1595-treated rats may be attributable, in part, to a selective increase in membrane GluR1 levels (Figure 4). GluR1 is a vital mechanism for both short and long-lasting adjustments in synaptic efficacy. Accordingly, the overall levels, phosphorylation status, and subcellular localization of GluR1 are closely regulated [61], [62]. While it is possible that TNF/TNFR1 signaling helps to suppress GluR1 at the transcriptional level, previous work showed that TNF modulates GluR trafficking to the plasma membrane [63], possibly in a TNFR1-dependent manner [64]. Clearly, aging and TNF-mediated differences in GluR1 transcriptional regulation and subcellular trafficking will require further consideration using synapse-specific tissue preparations (*i.e.* synaptosomes) and/or more targeted molecular approaches.

### Interaction between L-VSCCs and TNF during aging

Cognitive and synaptic alterations in aged animals, as well as numerous age-related diseases, are commonly linked to the dysregulation of Ca^2+^ [9], [48], [65], [66], [67], [68], [69]. Of the many mechanisms implicated in Ca^2+^ dysregulation, L-VSCCs are perhaps most consistently associated with changes in physiology and cognition in models of non-pathological aging. Increased neuronal L-VSCC activity in the hippocampus of aged rats, as first reported by Thibault and Landfield [13], underlies elevated Ca^2+^-induced Ca^2+^ release [70], [71], [72], augmented post-spike afterhyperpolarizations [73], impaired synaptic facilitation [41], and altered thresholds for induction of long-term potentiation (LTP) and LTD [37] during aging. Moreover, numerous studies have reported on the efficacy of L-VSCC antagonists on impaired cognitive function in aged rats [44], [60], [74].

There are several parallel observations that point to a strong interaction between L-VSCCs and TNF in the aging brain. L-VSCCs and TNF are both upregulated during aging and modulate key physiologic processes in the same direction, including LTP [75], [76], LTD [77] (also see Figure 3D and 3E), and behavioral acquisition [78], [79] (also see Figure 3A). But, more directly, TNF has been shown to potentiate L-VSCC activity in primary hippocampal neuronal cultures [11]. Based on these observations, we determined the necessity of TNF signaling in the regulation of L-VSCC activity in individual CA1 neurons of aged rats using the hippocampal “zipper” slice preparation and on-cell patch clamp recording. Consistent with a permissive effect of TNFR1 on L-VSCC function, we observed a significant reduction in L-VSCC activity in aged rats treated with XPro1595. The mechanistic basis for TNFR1 actions on L-VSCCs is unknown, but does not appear to involve an upregulation of the major L-VSCC pore-forming subunits (see Figure 5D and 5E), though an effect of XPro1595 on the subcellular trafficking of L-VSCCs cannot be ruled out. Previous work has identified the involvement of several critical signaling intermediaries in L-VSCC regulation during aging including the cAMP-dependent protein kinase [80], the protein phosphatase calcineurin [25], [81], and FK-506 binding proteins [82]. Each of these mechanisms is stimulated by and/or interacts with TNF in multiple cell types [83], [84], [85], [86], [87], [88], suggesting that the interaction between TNF and L-VSCCs in aged brain may be multifaceted.

### Cellular target of XPro1595: Neurons vs. Glia

As shown in Figure 2, XPro1595 caused a significant reduction in protein levels for the microglial marker, Iba-1, in aged rats (Figure 2). It seems likely that this observation is based on reduced microglial activation, rather than microglial cell loss or apoptosis, since TNF and/or TNFR1 tends to promote cell death, while TNFR1 blockade generally improves cell viability [1], [29], [89]. Similar to our findings, suppression of microglial activation with XPro1595 has been reported in primary murine microglial cultures [18] and also in a rodent model of Parkinson's disease [19]. Generally, drugs and/or genetic manipulations that reduce microglial activation also positively affect neuronal function and/or cognition in experimental models of aging and disease, probably by suppressing the release of numerous pro-inflammatory cytokines [90], [91], [92], [93], [94]. Like many other cytokine receptors, TNFRs are expressed on both neurons and glial cells [95]. Thus, it is unclear at this time whether the beneficial effects of XPro1595 on synaptic plasticity and cognition in aged rats are attributable to direct actions on neuronal receptors, or to indirect effects on microglia-based cytokine release, or both. Regardless, the results strongly suggest the TNF system plays a critical role in normal, as well as, pathological aging.

### Conclusion

The present study used a multi-disciplinary approach to investigate interactions among TNF signaling, synaptic function, and Ca^2+^ regulation during normal aging. Neuroinflammation, cognitive deficits, impaired plasticity, and increased L-VSCC activity were all ameliorated in aged rats following chronic treatment with the novel anti-TNF biologic XPro1595. The results suggest that TNF may drive age-related neurologic dysfunction through differential interactions with TNFR1 and TNFR2.
